# Implementation leadership: Confirmatory factor analysis and supervisor-clinician discrepancy in ratings on the Implementation Leadership Scale (ILS)

**DOI:** 10.1186/1748-5908-10-S1-A70

**Published:** 2015-08-20

**Authors:** Gregory A Aarons, Mark G Ehrhart, Lauren R Farahnak, Natalie Finn

**Affiliations:** 1 University of California, San Diego, La Jolla, CA 92093, USA; 2 San Diego State University, San Diego, CA, USA

## Introduction

In healthcare and allied healthcare settings, leadership that supports effective implementation of evidenced-based practices is critical. There is only one empirically validated measure to assess implementation leadership, the Implementation Leadership Scale (ILS). This study moves beyond testing the ILS with clinicians to assess the factor structure with clinical supervisors. Leader self-ratings can provide important insight into how leaders perceive their own abilities and behaviors, and assessing the discrepancies between leader self-ratings and follower ratings can be particularly useful in developing leaders' self-awareness, informing leadership development, and improving organizational functioning.

## Methods

Participants included 119 clinical supervisors and 441 clinicians. All participants completed the ILS with supervisors rating themselves and clinicians reporting on their own immediate supervisor. We conducted confirmatory factor analyses utilizing supervisor data, accounting for the nested data structure (i.e., supervisors nested within agency [k = 31]) and indicating a hypothesized second order factor structure. We then conducted t-tests to examine ILS scale score discrepancies between supervisor self-ratings and clinician ratings of their supervisors.

## Findings

Multilevel confirmatory factor analyses showed good fit to the hypothesized second order factor structure (CFI = 0.96, TLI, 0.95, SRMR = 0.05). First order factor loadings ranged from 0.85-0.92 for Proactive Leadership, from 0.93-0.96 for Knowledgeable Leadership, 0.88-0.94 for Supportive Leadership, and 0.83-0.96 for Perseverant Leadership, and second order factor loadings ranged from 0.74-0.95. Discrepancy analyses demonstrated that supervisors rated themselves significantly higher than clinicians on the Proactive (p < 0.05) and Supportive (p < 0.01) ILS subscales.

## Conclusions

The factor structure of the ILS is robust indicating its utility for both supervisor and clinician report. Discrepancies between supervisor and clinician ratings suggests that leadership training based on 360 degree assessments could be utilized to create development plans to improve the implementation and sustainment leadership knowledge, skills, and behaviors of supervisors with responsibilities for supporting evidence-based practices in the workplace.

